# The association between body mass index and skin problems in persons with a lower limb amputation: an observational study

**DOI:** 10.1186/s12891-021-04646-2

**Published:** 2021-09-09

**Authors:** Terezka S. Mollee, Pieter U. Dijkstra, Rienk Dekker, Jan H.B. Geertzen

**Affiliations:** 1 Department of Rehabilitation Medicine, University of Groningen, University Medical Center Groningen, PO box 30.001, Internal postal code CB40, 9700 RB Groningen, The Netherlands; 2 Department of Oral and Maxillofacial Surgery, University of Groningen, University Medical Center Groningen, Groningen, The Netherlands

**Keywords:** Amputation, Obesity, Body Mass Index, Body Weight, Skin, Prostheses, Rehabilitation

## Abstract

**Background:**

Obesity is common in persons with a lower limb amputation, an amputation can also lead to further weight gain. Data regarding the prevalence of obesity in the Dutch population with a lower limb amputation are lacking. Furthermore, the impact of obesity on skin problems of the residual limb and the need of prosthetic repairs is unknown. The aim of this study was to determine the prevalence of obesity in Dutch persons with a lower limb amputation and to investigate the relationship between body weight, body mass index and skin problems of the residual limb and the frequency of prosthetic repairs.

**Methods:**

A survey was performed among adults with a unilateral lower limb amputation due to any cause, and who are user of a prosthesis. The survey consisted of measurement of the subjects’ body height and weight, a questionnaire which assessed self-reported skin problems in the previous month and factors potentially associated with these skin problems, and assessment of the frequency of visits to the orthopedic workshop.

**Results:**

In total, 413 persons were enrolled. Of them, 39% (95 % confidence interval 35;44) were overweight and 28% (95% confidence interval 24;33) were obese. A total of 77% (95% confidence interval 73;81) reported one or more skin problems in the past month. Body weight and body mass index were neither associated with the presence of skin problems in general nor with the number of prosthetic repairs. Persons with severe skin problems had a slightly lower body mass index (26.6 kg/m^2^ vs. 28.0 kg/m^2^, *p* = 0.012). Persons with skin problems were younger than those without (difference in means 6.0 years (95% confidence interval 3.0;8.9)).

**Conclusions:**

Our findings show that obesity is common in the Dutch ambulant population with a lower limb amputation, with a prevalence being higher than in the general Dutch adult population. However, its negative impact on the presence of skin problems and the frequency of prosthetic repairs may be limited.

## Background

Weight gain after a lower limb amputation (LLA) is a common phenomenon. The majority of people undergoing a dysvascular amputation, the most important reason for amputation, are overweight (body mass index (BMI) ≥ 25 kg/m^2^) or obese (BMI ≥ 30 kg/m^2^) prior amputation and continue to gain weight in the year after amputation [1–4]. The prevalence of obesity increases with a more proximal level of amputation [5].

For individuals with a LLA who are overweight or obese it is important to have a suitable prosthesis that can withstand the forces caused by the body weight. A study from the United Kingdom showed that about 14 % of persons with a LLA have a body weight over 100 kg, the general weight limit for prosthetic components, and in 3 % the body weight exceeds the weight limit of the prosthesis, thus risking material failure and falling [6]. The number of prosthetic repairs increases with body weight [7]. It has been suggested that obesity and impaired wound healing after amputation and problems with prosthetic fitting are associated [5].

Several possible determinants of skin problems of the residual limb have been studied before. Older persons with a LLA may face a lower risk of developing skin problems than their younger counterparts, probably due to a decreased level of activity and less use of the prosthesis [8]. Furthermore, it has been suggested that older, less active people with a LLA need less prosthetic repairs than younger, active people [7, 9]. Diabetes and/or peripheral arterial disease (PAD) as reason(s) for amputation are also associated with less frequent skin problems [8].

Studies concerning the association between BMI and functional outcome measures in persons with a LLA are scarce. One study pointed out that BMI is no independent predictor of overall prosthetic usage, 36-month ambulation, survival and independent living status in persons with a dysvascular amputation [10]. Another study concluded that obesity does not affect inpatient amputation rehabilitation outcomes [11]. These findings suggest that BMI and especially obesity plays no important role in functional benefit post-amputation.

Data regarding the prevalence of obesity in the Dutch population with a LLA are missing. Although previous studies showed that BMI is not associated with functional outcome, the role of BMI in the presence of skin problems and the frequency of prosthetic repairs has not been investigated before. Better understanding of the influence of obesity on these issues may help improve the monitoring and treatment of this vulnerable population, and potentially address the importance of primary and secondary prevention of obesity.

The primary objective of this study was to determine the prevalence of obesity among persons with a LLA. The secondary objective was to analyse a possible association between body weight, BMI and skin problems of the residual limb and the frequency of prosthetic repairs within this population.

## Methods

### Study design

A survey was conducted by measuring the participants’ body weight and height at the orthopedic workshop, combined with the use of a questionnaire which assessed the presence of skin problems of the residual limb and factors potentially associated with these skin problems. Data were obtained from persons with a LLA who received their prosthesis through two large orthopedic workshops, ‘OIM Orthopedie’ and ‘Orthopedietechniek’. The study was approved by the Medical Ethical Committee of the University Medical Center Groningen and all participants provided written informed consent.

### Participants

Inclusion criteria were: (1) age ≥ 18 years; (2) unilateral LLA due to any cause, ranging from ankle disarticulation to hip disarticulation; (3) date of amputation ≥ 1 year before inclusion; (4) user of a prosthesis; (5) able to stand on a personal scale with a prosthesis, if necessary using minimal support. Exclusion criteria were: (1) user of an osseointegration prosthesis; (2) additional upper extremity amputation; (3) insufficient knowledge of the Dutch language and/or insufficient cognitive skills to fill in the questionnaire.

### Materials

The questionnaire was based on one used previously [8, 12–14]. It included 54 questions providing information about: participant, amputation and prosthesis, hygiene of the prosthesis and residual limb, level of physical activity, and self-reported skin problems in the month prior to filling in the questionnaire. The participants’ body weight and height were measured by certified prosthetists using a personal scale (Tefal PP1220 Premio) and a ruler which was fixed to a wall. Participants were asked to stand on the scale wearing their prosthesis and underwear only. The weight of the prosthesis, measured separately, was subtracted. The height of participants was measured while standing against the wall without wearing shoes. If it was unsafe to stand, the height noted in the passport was recorded.

### Data collection

Data were collected between December 12th 2017 and July 31st 2019. The majority of persons with a LLA received an invitation letter including the questionnaire by mail at least 1 week prior to their appointment at the orthopedic workshop. If they agreed to participate, they signed the attached informed consent form and provided their contact data. Most participants filled in the questionnaire at home and brought it to their appointment, where measurements were performed and additional data were collected. These data included the number of visits to the orthopedic workshop in the past year and the number of visits for fitting a new prosthesis in that period. The number of visits to the orthopedic workshop was used as a proxy for the frequency of prosthetic repairs.

Some persons with a LLA had an appointment less than 1 week ahead. They were invited to participate during their visit by handing out the letter, questionnaire and a prepaid return envelope. If they agreed to participate, the measurements were carried out during their visit. Finally, potential participants were recruited during an annual assembly for people with an amputation and/or congenital limb reduction. The same procedures were followed, except that TM (first author) performed the measurements and participants were allowed to keep their clothes on. A clothing adjustment of 0.8 kg for women and 1.2 kg for men was used [15].

Nonparticipants were asked to fill in a response form which provided information about their sex, date of birth and amputation level.

### Data entry

Data were entered into a database. If answers were missing, the participant was contacted by telephone or the questionnaire was sent back with the request to provide the missing information. If participants mentioned more than one reason for amputation and/or had not filled in the exact date of the amputation, we followed the procedures previously described [8].

### Body mass index

The adjusted BMI represents the pre-surgical BMI, which is adjusted for the mass of the lost limb [1, 16–18]. The adjusted body weight (W_a_) was calculated with the formula: W_a_ = W_m_ / (1 – *p*), where W_m_ is the measured body weight and *p* is the proportion of body weight that is missing due to amputation [1, 11]. The proportions of body weight for different body segments of the lower extremity are based on calculations of Durkin [18]. Based on expert opinion, estimations are that 100 % of the foot and 50 % of the leg is removed in trans-tibial amputation, and 100 % of the foot and leg and 40 % of the thigh is removed in trans-femoral amputation [1]. When these estimations are combined with the mentioned body proportions, final percentages of removed body weight per amputation level are: 1.32 % for ankle disarticulation, 3.44 % for trans-tibial amputation, 5.56 % for knee disarticulation, 10.62 % for trans-femoral amputation and 18.2 % for hip disarticulation. Regarding rotationplasty, we estimated that the percentage of missing body weight is equal to the percentage for knee disarticulation. Body proportions from Durkin are categorized by sex and age groups 19–30 and > 55 years. Because proportions for the category 30–55 years were lacking, we extrapolated them to age groups 19–40 and > 40 years as done previously [11].

A BMI of < 18.5 kg/m^2^ corresponded with underweight, a BMI of 18.5–24.9 kg/m^2^ with normal weight, a BMI of 25–29.9 kg/m^2^ with overweight, and a BMI of ≥ 30 kg/m^2^ with obesity.

### Sample size estimation

We estimated that 70 % of persons with a LLA would be overweight or obese, to estimate this percentage with a ± 2.5 % precision a sample size of 1350 was needed.

### Statistical analysis

A selection of ordinal variables was dichotomized for a more concise representation of the characteristics: duration of use of the prosthesis (< 12 or ≥ 12 h per day), walking distance outdoors (< 500 or ≥ 500 m per day), use of the prosthesis for walking indoors (< 50 or ≥ 50 % of time) and use of a walking aid (yes or no). The outcome measures regarding skin problems were categorized into skin problems in general, severe skin problems and number of skin problems. The participant was asked if the following skin problems were either present or not: abrasion, blisters, cold skin, corn/callus, crusts, existing wound, pimples, profuse sweating, redness of skin > 1 min after removing prosthesis, swollen skin, warm skin, white/blue skin. The participant was also asked about the perceived severity of these skin problems, defined as the degree of the overall negative impact on the use of the prosthesis. Considering the presence of an itching, prickly, sensitive or painful skin, a separate 4-point rating scale was used in the questionnaire to identify the severity. Dichotomization was applied for the items shown in Table 1.

**Table 1:** Dichotomization of items in questionnaire about skin problems

Question	Possible answers	Dichotomization
Overall negative impact of skin problem on use of prosthesis	none, limited high, very high	not severe severe
Presence of itching, prickly, sensitive or painful skin	none limited, high, very high	absent present
Severity of itching, prickly, sensitive or painful skin	none, limited high, very high	not severe severe

Factors analysed for a possible association with (severe) skin problems and prosthetic repairs were body weight, BMI, age, walking distance outdoors, use of a walking aid (as a proxy for activity level) and reason for amputation. Pearson correlation coefficients were calculated for the analysis of normally distributed interval variables. Spearman’s rho correlation coefficients were calculated for ordinal data or not normally distributed data. Independent sample t tests were used for the analysis of continuous data. Nonparametric tests (Kruskal-Wallis tests, Mann-Whitney U tests and chi-squared tests) were used for the analysis of nominal and quantitative data. A *p*-value of ≤ 0.05 was considered statistically significant. SPSS 23 was used to perform the statistical analysis.

## Results

### Participants and nonparticipants

**Fig. 1:**
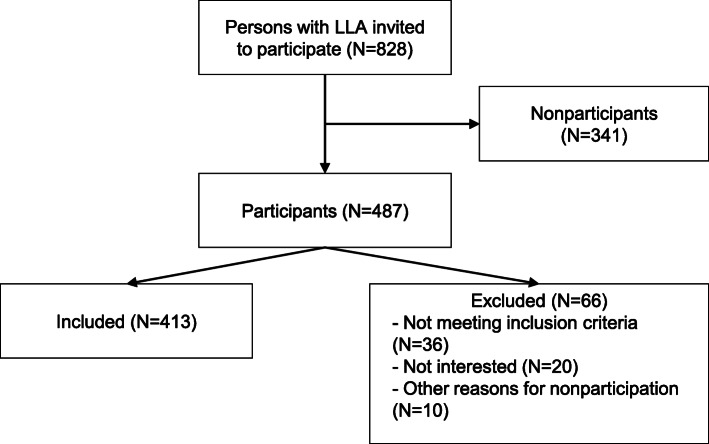
Flowchart of study sample and in- and excluded participa nts

A total of 828 persons with a LLA were invited to participate (Fig. 1). Of the 487 people who responded, 421 agreed to participate (51 %); 66 persons who did not want to participate sent back their response form. Eight persons filled in the questionnaire, but were excluded from analyses because the date of amputation was less than 1 year ago, leaving 413 participants for statistical analyses. No statistically significant differences were found between participants and nonparticipants (Table 2).

**Table 2:** Descriptive statistics of participants and nonparticipants

Characteristics	Participants (***N*** = 413)	Nonparticipants (***N*** = 66)	***p***
Mean age ±SD (y)	62.3 ±14.9	64.3 ±11.9	0.320
Sex (%)	413 valid cases	65 valid cases	
Men	290 (70%)	44 (68%)	0.680*
Women	123 (30%)	21 (32%)	
Amputation level (%)	409 valid cases	57 valid cases	
Ankle disarticulation	5 (1%)	0 (0%)	0.235**
Trans-tibial amputation	234 (57%)	26 (46%)	
Knee disarticulation	44 (11%)	7 (12%)	
Trans-femoral amputation	116 (28%)	24 (42%)	
Hip disarticulation	6 (2%)	0 (0%)	
Rotationplasty	4 (1%)	0 (0%)	
BMI (kg/m^2^)	386 valid cases		
Mean BMI ±SD (95% CI)	27.5 ±5.0 (27.0−28.0)		
BMI category (%)	386 valid cases		
Underweight (<18.5 kg/m^2^)	7 (2%)		
Normal weight (18.5-24.9 kg/m^2^)	119 (31%)		
Overweight (25.0-29.9 kg/m^2^)	152 (39%)		
Obesity (≥30 kg/m^2^)	108 (28%)		
Body weight (%)	380 valid cases		
<100 kg	330 (87%)		
≥100 kg	50 (13%)		
Median time since amputation (y, IQR)	400 valid cases		
	12.4 (3.2;30.7)		
Reason for amputation (%)	409 valid cases		
Trauma	159 (39%)		
Diabetes and/or PAD	114 (28%)		
Other	47 (12%)		
Malignancy	40 (10%)		
Infection	38 (9%)		
Congenital	11 (3%)		
Comorbidity (%)	405 valid cases		
None	207 (51%)		
Duration use of prosthesis (%)	405 valid cases		
0-12 hours/day	142 (35%)		
≥12 hours/day	263 (65%)		
Walking distance outdoors (%)	404 valid cases		
<500 meters/day	219 (54%)		
≥500 meters/day	185 (46%)		
Time walking with prosthesis indoors (%)	407 valid cases		
<50%	105 (26%)		
≥50%	302 (74%)		
Use of walking aid (%)	407 valid cases		
Yes	224 (55%)		
Any skin problem in month prior to completing questionnaire (%)	413 valid cases		
Yes	319 (77%)		
Severe skin problem in month prior to completing questionnaire (%)^a^	397 valid cases		
Yes	125 (32%)		
Skin problems in period >1 month prior to completing questionnaire (%)	400 valid cases		
Yes	250 (63%)		

^a^Skin problem with a negative impact on use of the prosthesis and/or a high or very high degree of discomfort due to an itching skin, sensitive skin, prickly skin and/or painful skin (using a 4-point scale: none, limited, high, very high)

^*^Pearson Chi-Square = 0.170

^**^Pearson Chi-Square = 6.548, exact procedure

### Participant characteristics

Most participants were male (70 %) and the mean age was 62.3 ± 14.9 years (Table 2). The median time since amputation was 12.4 years (interquartile range (IQR) 3.2;30.7). The most common level of amputation was trans-tibial (57 %). Most frequent reasons for amputation were trauma (39 %) and diabetes and/or PAD (28 %). A total of 51 % had no (relevant) comorbidity, 24 % had diabetes and 17 % had vascular disease. A minority of 31 % were employed, and 21 % were smoker. Of the participants, 39 % (95 % confidence interval (CI), 35;44) were overweight, 28 % (95 % CI, 24;33) were obese and 13 % (95 % CI, 10;17) had a body weight over 100 kg. A majority used the prosthesis 12 or more hours per day (65 %) and walked less than 500 m per day (54 %).

### Skin problems

In total, 77 % (95 % CI, 73; 81) reported one or more skin problems in the previous month and 32 % (95 % CI, 27;−36) mentioned a severe skin problem. Participants with skin problems were on average 6.0 years younger than participants without skin problems (Table 3). Participants with severe skin problems had a lower BMI than participants without severe skin problems (26.6 kg/m^2^ vs. 28.0 kg/m^2^, *p* = 0.012). Participants with severe skin problems were also significantly younger than those without (difference in means 5.5 years, *p* = 0.001). No significant association was found between the presence of skin problems and reason for amputation (*p* = 0.196 for any skin problems; *p* = 0.091 for severe skin problems).

**Table 3:** Association between skin problems in previous month and body weight, body mass index and age

	No	Yes			
	Mean ±SD	Mean ±SD	Difference	95% CI	***p***
**Any skin problem**					
Body weight (kg) (*N* = 388)	80.8 ±14.9	80.7 ±19.2	0.1	-3.7;3.9	0.958
BMI (kg/m^2^) (*N* = 386)	28.2 ±3.8	27.3 ±5.3	5.3	-0.1;1.9	0.074
Age (y) (*N* = 387)	66.9 ±11.4	60.9 ±15.6	6.0	3.0;8.9	<.001
**Severe skin problem**					
Body weight (kg) (*N* = 376)	82.1 ±18.5	79.0 ±17.5	3.2	-0.8;7.1	0.119
BMI (kg/m^2^) (*N* = 376)	28.0 ±5.1	26.6 ±4.9	1.4	0.3;2.5	0.012
Age (y) (*N* = 376)	63.6 ±14.3	58.0 ±15.4	5.5	2.3;8.7	0.001

Participants with severe skin problems used a walking aid more frequently than participants without severe skin problems (62 % vs. 51 %, Pearson Chi-Square 4.410, *p* = 0.036). Post-hoc analysis showed that participants with an amputation due to diabetes and/or PAD reported a significantly lower number of skin problems than participants with an amputation due to trauma (median 2 vs. 3, *p* = 0.01).

### Prosthetic repairs

No association was found between body weight, BMI, age or walking distance outdoors and the number of visits to the orthopedic workshop (Table 4). The analysis included an outlier with 80 visits to the orthopedic workshop in the previous year; after exclusion of this outlier none of the outcomes did change relevantly. Participants who used a walking aid visited the orthopedic workshop more frequently than those without a walking aid (*p* = 0.043 for total number of visits). The number of visits was also associated with the reason for amputation, with amputation due to malignancy resulting in the most visits (*p* = 0.005 for total number of visits). The number of visits increased with the number of skin problems (Spearman’s rho 0.184, *p* < 0.001).

**Table 4:** Association between different parameters and the number of visits to the orthopedic workshop

Determinants	Total number of visits to orthopedic workshop in past year (Spearman’s rho)	*p*
Body weight (kg) (*N* = 381)	-0.022	0.663
BMI (kg/m^2^) (*N* = 380)	-0.042	0.416
Age (y) (*N* = 381)	-0.036	0.479
Walking distance outdoors (m/day)^a^ (*N* = 375)	-0.017	0.741
Use of walking aid (*N* = 379)	Median (IQR)	
Yes	6 (3;11)	0.043
No	5 (3;8)	
Reason for amputation (*N* = 380)		
Trauma	4 (2;7)	0.005
Diabetes and/or PAD	6 (3;11)	
Other	6 (4;12)	
Malignancy	7 (3;14)	
Infection	5 (3;9)	
Congenital	4 (1;5)	
	Number of visits to orthopedic workshop in past year, visits for fitting new prosthesis excluded (Spearman’s rho)	
Body weight (kg) (*N* = 378)	-0.002	0.970
BMI (kg/m^2^) (*N* = 377)	-0.008	0.884
Age (y) (*N* = 378)	-0.059	0.254
Walking distance outdoors (m/day)^a^ (*N* = 372)	-0.030	0.566
Use of walking aid (*N* = 376)	Median (IQR)	
Yes	4 (2;8)	0.007
No	4 (1;7)	
Reason for amputation (*N* = 377)		
Trauma	3 (2;6)	0.009
Diabetes and/or PAD	4 (2;8)	
Other	5 (3;9)	
Malignancy	6 (2;12)	
Infection	5 (2;9)	
Congenital	2 (1;4)	

^a^Walking distance outdoors: <100 m, 100–200 m, 500 1000 m, > 1000 m

## Discussion

A majority of Dutch persons with a LLA were overweight or obese and most participants reported skin problems of the residual limb. Body weight and BMI were not associated with skin problems in general. Participants who reported severe skin problems had a lower BMI and were younger than participants who did not report severe skin problems. No association was found between body weight, BMI and the frequency of prosthetic repairs.

Because the presence of an above normal BMI is associated with a higher risk of developing various health problems, clinicians should pay attention to the BMI of a person with a LLA and discuss possible measures to reduce weight. However, the results of our study show that overweight or obesity may not be a risk factor in the development of skin problems and the need for prosthetic repairs.

The prevalence of obesity (28 %) in the studied population of Dutch persons with a LLA was higher than in the general Dutch adult population (15 %), the prevalence of overweight (39 %) was similar to that of the general Dutch adult population (36 %) [19]. Previously, high prevalences of overweight and obesity among persons with a unilateral or bilateral LLA have been reported (82.5 % up to 84.0 %), with obesity based on BMI found in 29.5 % up to 50.7 % of cases [1, 20, 21].

The prevalence of skin problems in our study (77 %) was higher than in other studies (34 % up to 74 %) [8, 22–25]. This difference could be explained by variations in operationalization of skin problems and method of assessment (self-reported vs. assessment by a physician). The influence of body weight and BMI on the presence of skin problems was less outspoken than we anticipated, this finding could have several reasons. The residual limb of an overweight person with a LLA may consist of relatively more fat tissue distributing pressure better over bony prominences. Additionally, overweight persons with a LLA might be physically less active than those with a healthy weight, leading to less pressure and shear and stress forces and subsequently less skin problems [8]. Further, overweight or obese persons more often have comorbidity, which may lead to stricter post-amputation monitoring and skin care. Persons with severe skin problems had a lower BMI than persons without severe skin problems, but both groups were in the overweight category and the clinical relevance of this finding is doubtful.

We found an inverse association between age and skin problems. In an earlier study, older age was also protective for skin problems [8]. Similarly, employment and use of no or a light type of walking aid were associated with skin problems [22]. Younger persons possibly have a higher level of physical activity, leading to more skin problems [8].

Body weight and BMI were not associated with the number of prosthetic repairs. A possible explanation is that the number of visits to the orthopedic workshop was not an adequate proxy for the number of prosthetic repairs. Another possibility is that during the process of prescribing a prosthesis, body weight limits were already taken into account consistently, leading to less frequent failure of the prosthesis. Since a prosthesis is designed to carry a person’s body weight without taking height into account, BMI was not expected to play a significant role. In total, 13 % of participants had a body weight over 100 kg, which is similar to other findings [6].

An association was found between the use of a walking aid and the number of prosthetic repairs; perhaps a walking aid was used because of problems with prosthetic fitting. Reason for amputation and frequency of prosthetic repairs were associated as well, but the underlying (probably multifactorial) mechanisms were not further analysed in this study.

### Study limitations

We were not able to include our calculated sample size of 1350 participants. However, with the inclusion of 413 participants we reached a precision of ± 4.6 %. Also, selection bias may have occurred. In the general population of persons with a LLA, diabetes and/or PAD are the leading cause of amputation [4, 26]. In contrast, trauma was the most reported reason for amputation in our study. Still, the results may be applicable to the outpatient population with a LLA that has a lower mortality rate and has to deal with (often chronic) skin problems. Additionally, it is possible that persons with a healthy BMI were more willing to participate than persons with an above normal BMI. This could have resulted in an underestimation of the prevalence of overweight and obesity. Similarly, persons with skin problems may have been more likely to take part in the study, leading to an overestimation of the prevalence of skin problems. Another limitation is the fact that skin problems were self-reported and no physician was involved to examine the skin condition of the participants who mentioned skin problems. The design of the study was not suitable for identifying the etiology of the skin problem, for example if it was caused by a reaction to pressure on the residual limb or a reactivation of dermatoses that were already present (known as Koebner’s phenomenon). Nevertheless, the number of participants with skin problems based on self-report is in the same range when compared to data collection through clinical observation [14]. Moreover, the questionnaire made it possible to use a time window of 1 month in which a skin problem could be reported. This window makes it easier to identify skin complaints that can fluctuate over time and could be missed at the moment of assessment by a physician. In order to draw firmer conclusions, future studies with larger sample sizes and clinical assessment of skin problems are needed.

## Conclusions

Two thirds of Dutch persons with a LLA have an above normal BMI, with almost one third being obese. No association was found between body weight, BMI and skin problems of the residual limb in general and the frequency of prosthetic repairs. Still, we recommend careful assessment of the BMI in this population, as obesity has a well-known negative impact on general health.

## Data Availability

The datasets used and/or analysed during the current study are available from the corresponding author on reasonable request.

## Abbreviations

BMI: Body mass index
CI: Confidence interval
IQR: Interquartile range
LLA: Lower limb amputation
PAD: Peripheral arterial disease
SD: Standard deviation
Y: Years
